# Laryngeal chondritis as a differential for upper airway diseases in German sheep

**DOI:** 10.1186/s13028-020-0510-0

**Published:** 2020-03-04

**Authors:** Wencke Reineking, Teresa Maria Punsmann, Matthias Gerhard Wagener, Jutta Verspohl, Martin Ganter, Wolfgang Baumgärtner, Christina Puff

**Affiliations:** 1 Department of Pathology, University of Veterinary Medicine Hannover, Foundation, Bünteweg 17, 30559 Hannover, Germany; 2 Clinic of Swine, Small Ruminants and Forensic Medicine, University of Veterinary Medicine Hannover, Foundation, Bischofsholer Damm 15, 30173 Hannover, Germany; 3 Institute for Microbiology, University of Veterinary Medicine Hannover, Foundation, Bischofsholer Damm 15, 30173 Hannover, Germany

**Keywords:** Dyspnea, Laryngeal chondritis, Ovine

## Abstract

**Background:**

Ovine laryngeal chondritis is a rare entity of sheep in the USA, Great Britain, New Zealand and Iceland, but has not been reported in Germany so far. Here, two German cases are reported.

**Case presentation:**

Two rams showed severe and progressive signs of dyspnea. Endoscopically, a severe bilateral swelling of the larynx was identified in both rams. Due to poor prognosis and progression of clinical signs one ram was euthanized, while the other ram died overnight. In both cases, a necrosuppurative laryngitis and chondritis of arytenoid cartilages was found at necropsy. *Fusobacterium necrophorum* and *Streptococcus ovis* were isolated from the laryngeal lesion in one animal.

**Conclusions:**

This is the first report of ovine laryngeal chondritis in continental Europe. This entity should be considered a differential diagnosis for upper airway disease in sheep.

## Background

Airway diseases are a common problem in sheep leading to decreased animal welfare and economic losses [1, 2]. Respiratory signs of sheep are often caused by multiple factors including a variety of infectious agents [1–3]. The course of disease, morbidity and mortality varies depending on severity of lesions and physical condition of the flock and individual [1]. Especially in young sheep, respiratory tract infections are an important cause of morbidity and mortality [3]. Bacterial pneumonia in sheep is mainly caused by *Pasteurella multocida, Mannheimia haemolytica* or *Histophilus somni* [4]. Often a primary pulmonary injury by viral or mycoplasmal infections as well as by inhalation of toxic or irritating compounds impairs the defense mechanisms of the lung, thereby facilitating the colonization by bacteria [4].

Laryngitis often concurs lesions of the upper and lower respiratory tract but also occurs separately as an independent disease [4]. Ovine laryngeal chondritis is characterized by chronic inflammatory changes of the arytenoid cartilages [5]. The following cases are the first descriptions of this entity in Germany and in mainland Europe.

## Case presentation

Two rams (cases 1 and 2) were referred to the Clinic of Swine, Small Ruminants and Forensic Medicine of the University of Veterinary Medicine Hannover, Foundation, Germany due to severe respiratory problems in October 2017 and February 2019, respectively. Case 1 was a 7-month-old crossbreed ram with severe progressive stridor and dyspnea. Case 2 was a 2.5-year-old Texel ram, which was noticed by the owner due to severe respiratory problems for three days. The latter animal received an initial antibiotic treatment (benzylpenicillin-natrium, neomycinsulfate, Neopen 200/150 mg/mL, Intervet Deutschland GmbH, Unterschleißheim, Germany) that did not improve the clinical signs and therefore the ram was hospitalized for diagnostics and treatment. Clinical examination of both animals revealed an increased respiratory rate (case 1: 40 breaths per minute; case 2: 78 breaths per minute) with intensified abdominal breathing. A severe in- and expiratory laryngeal stridor was present. The heart rate was 48 beats per minute (case 1) and 108 beats per minute (case 2), respectively. The ram of case 1 displayed moderately injected episcleral vessels and slightly reddened conjunctivae as sings of circulatory stress. Case 2 presented with a stretched position of the neck and head of the ram due to severe respiratory distress accompanied by cyanosis of the conjunctivae and gingiva indicative of severe hypoxia. Palpation of the larynx revealed a slight enlargement in case 1. When light pressure was applied to the larynx, the first ram became hypoxic. After the pressure was released, a coughing attack was observed. In case 2 the larynx was symmetric in palpation, but manipulation of the larynx worsened the breathing considerably.

Blood smear evaluation revealed normal ratios of leukocytes in case 1 and a mild leukocytosis in the second ram (leukocytes: 12.6 G/L [reference range: 4–12 G/L]). The hematocrit was within normal range in both cases.

**Fig. 1:**
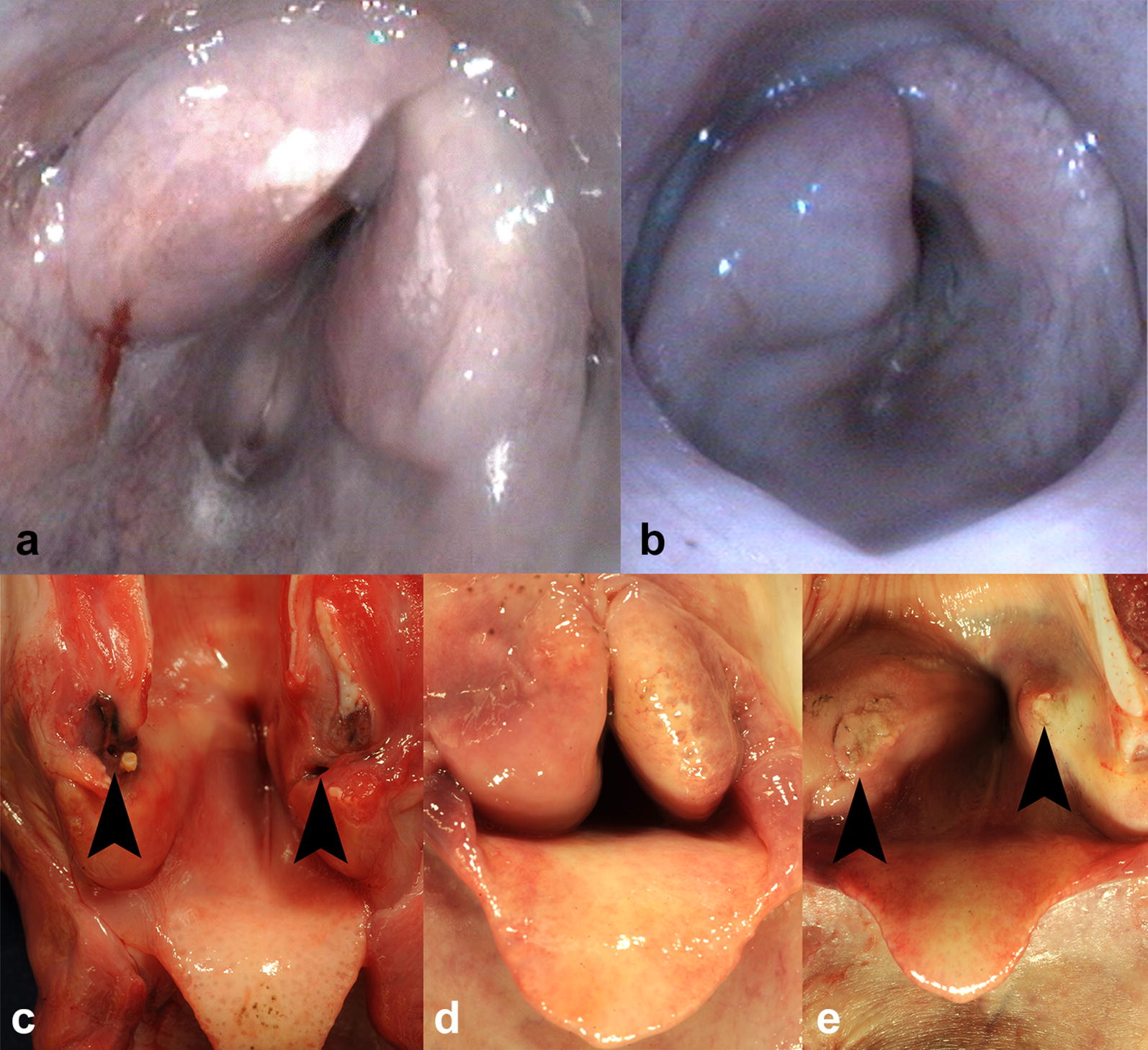
Larynx, sheep. **a** Endoscopy at maximal inspiratory opening: a bilateral severe symmetric swelling of the larynx with narrowing of laryngeal cavity can be seen in case 1. **b** Severe swelling of the larynx with constriction of the glottis is present during endoscopy in case 2. **c** Gross pathology, case 1: Bilaterally within the arytenoid processes dark brown oval ulcers with attached exudate (arrowheads) are present. **d** Necropsy confirmed the laryngeal swelling and constriction of the glottis in case 2. **e** Case 2: Bilaterally, raised lesions are found in the mucosa of the arytenoid cartilages (arrowheads)

Endoscopically, a severe bilateral symmetric swelling of the larynx with obstruction of the laryngeal cavity was observed in the first ram (Fig. 1a). For initial treatment of the first ram, meloxicam was administered subcutaneously (0.5 mg/kg; Melosolute® 20 mg/mL, CP-Pharma Handelsgesellschaft mbH, Burgdorf, Germany). After detection of laryngeal swelling the animal was treated with dexamethasone (Dexamethason® 4 mg/mL, Vetoquinol GmbH, Ismaning Germany). As the general condition of the ram worsened in the following days it was euthanized.

Similar to the first case, a swelling of the larynx was suspected in the second ram leading to an immediate treatment with 20 mg dexamethasone (Dexamethason® 4 mg/mL, Vetoquinol GmbH) intravenously. Despite this treatment the condition of the ram worsened. To prevent suffocation, a tracheotomy was performed and a tube was inserted into the trachea. After breathing was ensured the upper airways were examined endoscopically. This revealed a narrowed glottis caused by swelling of the mucous membranes of the right arytenoid cartilage (Fig. 1b). 20 mg dexamethasone were directly applied onto the glottis endoscopically. Furthermore, an antibiotic treatment with oxytetracycline (20 mg/kg, Ursocyclin® 10% pro inj. 100 mg/mL, MEDISTAR Arzneimittelvertrieb GmbH, Ascheberg, Germany) was started. After about one hour the ram was able to breathe without any problems through the tube and circulation state and general condition normalized within the following two hours. Despite this positive development the ram died overnight.

Both rams were necropsied at the Department of Pathology. Macroscopically, the larynx of case 1 showed a severe diffuse symmetric edematous swelling that caused a nearly complete obstruction of the laryngeal cavity. Bilaterally, the mucosa over the arytenoid cartilages had dark brown ulcers, 0.5 cm in diameter. These defects extended into the cartilage. Bilaterally within the arytenoid cartilages, a 0.5 × 1 × 1 cm sized cavitation filled with yellowish, viscous, opaque material was present. Similar exudate covered the surface of the mucosal lesions (Fig. 1c). In addition, a moderate multifocal suppurative bronchopneumonia was detected in the right cranial and medial pulmonary lobes involving about 20% of the lung parenchyma.

In case 2 similar lesions were detected. The larynx showed a severe diffuse bilateral asymmetric edematous swelling with nearly complete closure of the laryngeal cavity (Fig. 1d). Bilaterally, the mucosa over the arytenoid cartilages had raised nodules with mild multifocal ulcerations, 1 cm in diameter (Fig. 1e). Within the right arytenoid cartilage, a cavitation sized 0.8 × 0.8 × 1 cm, was present that was filled with a yellowish, flocculent, opaque material. Additionally, a raised, round, centrally ulcerated mass sized 5 × 5 × 2 cm, was present within the skin of the forehead.

Tissue samples from both cases were collected at necropsy, fixed in 10% neutral buffered formalin and routinely processed. For staining procedures, 2 µm sections of paraffin-embedded tissues were prepared, deparaffinized and rehydrated. Hematoxylin–eosin, Safranin O, Gram, Azan and Masson–Goldner stains as well as Periodic Acid Schiff (PAS) reaction were performed using standard staining protocols [6]. Alcian blue staining was carried out at pH 2.5 according to a standard protocol [6].

**Fig. 2:**
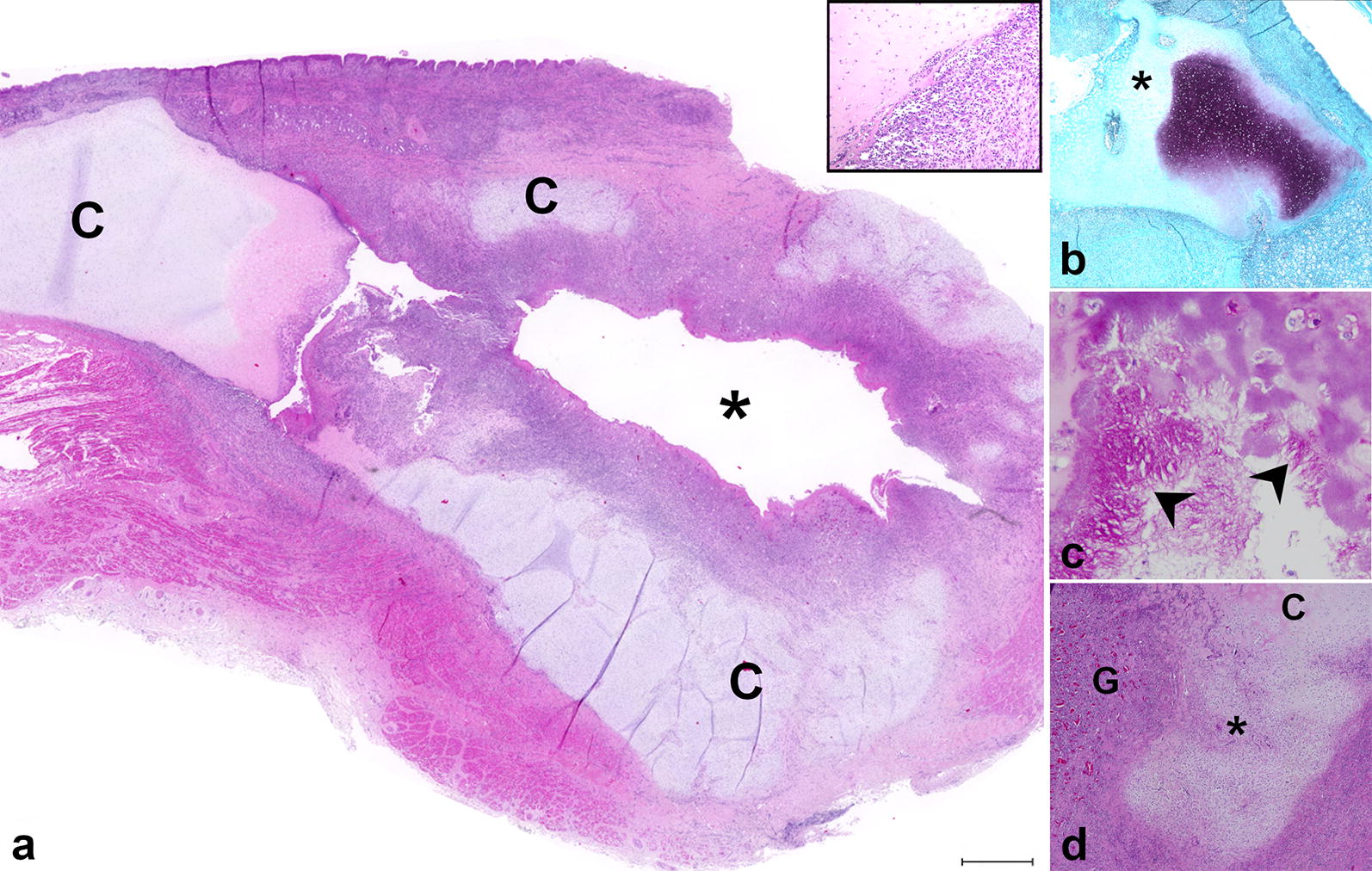
Larynx, sheep, Case 2. **a** Cross section of the larynx shows a large central defect within the arytenoid cartilage (C) with an inflammatory cell infiltration within the cavitation (insert) and surrounding tissue and loss of cartilage (asterisk). H&E. Bar: 1 mm. **b** Loss of cartilage is confirmed by loss of stainability (asterisk). Safranin-O. **c** Numerous filamentous PAS-positive bacilli were present at the cartilaginous surface (arrowhead). PAS reaction. **d** Pre-existing cartilage (C) is replaced by highly vascularized granulation tissue (G) surrounding large areas of necrotic cartilage (asterisk). H&E

Histologically, the arytenoid cartilages of both rams showed morphologically similar lesions but of varying severity. Case 1 presented bilateral cavitations within the arytenoid cartilages (Fig. 2a), which communicated with the laryngeal cavity. The cavitation and the surrounding cartilaginous, submucosal, glandular and muscular tissues were infiltrated by numerous inflammatory cells. The cellular infiltrates included high numbers of viable and degenerated neutrophils and fewer macrophages, lymphocytes and plasma cells. Additionally, moderate amounts of cellular debris were present mainly within the cavitation and rarely at the mucosal surface. The arytenoid cartilage showed numerous shrunken, hypereosinophilic chondrocytes and empty lacunae as well as a loss of basophilia, interpreted as necrosis. Furthermore, the cartilage surface lining the cavitation was irregularly shaped with clefts and fragmentation of cartilaginous matrix (Fig. 2a). The luminal surface of the cavitation was covered by high numbers of 0.2 × 7–10 µm sized basophilic filamentous bacteria and fewer approximately 1 µm in diameter sized cocci. The filamentous bacteria were also present within underlying parts of affected cartilaginous matrix. Accompanying the intracartilaginous cellular infiltrate, granulation tissue was observed.

Histochemically, the loss of arytenoid cartilage was confirmed by Alcian blue, Masson–Goldner and Safranin O stains. Intralesionally, the arytenoid cartilages revealed a decreased stainability by Alcian blue and Safranin O (Fig. 2b). In contrast, the staining intensity by Masson–Goldner was increased within the cartilaginous lesions.

At the inner surface of the cavitation and within the surrounding arytenoid cartilage abundant PAS positive, Gram-negative filamentous bacteria, arranged in chains were found (Fig. 2c). The Azan stain confirmed the presence of collagen fibers within the granulation tissue. Gram-positive coccoid bacteria were detected adjacent to necrotizing cartilaginous lesions. In Case 2, less pronounced cartilaginous necrosis and inflammation with an increased amount of granulation tissue were found (Fig. 2d). PAS reaction and Gram stain did not reveal any intralesional bacteria.

Additional histological findings in case 1 include a moderate suppurative bronchopneumonia partly with abscess formation in the right cranial and medial pulmonary lobes, a mild sinus histiocytosis in the pulmonary lymph nodes and a mild multifocal meningeal melanosis.

Additionally, in case 2, an ulcerated squamous papilloma was found on the forehead as an incidental finding.

Samples of the laryngeal lesions were submitted to the Institute of Microbiology to examine bacterial involvement by bacterial culture. Samples were processed and grown bacteria were differentiated up to species level regarding routine protocols. Microbiologically*, Fusobacterium necrophorum, Porphyromonas *sp*.* and *Streptococcus ovis* were isolated in large number from the laryngeal lesions of case 1. In addition, *Clostridium septicum* was isolated in moderate number in this sample.

Microbiologically, no bacterial growth was evident within laryngeal tissue in case 2. However, a negative impact of the antibiotic pre-treatment on bacterial growth has to be taken into account.

## Discussion and conclusions

The examination of the two rams revealed a chronic laryngitis with necrosis of the arytenoid cartilages and ulceration of the overlying mucosa. Laryngeal chondritis is a rare entity of sheep that has been reported in Great Britain [7], New Zealand [8–10], North America [5, 11] and Iceland [12] but it has not been reported in Germany yet. The disease occurs in both sexes and all ages but lambs and yearlings are affected predominantly. One reason for the predominance of affected younger animals may be due to the smaller size of the *rima glottidis* [7]. The disease is characterized by a low morbidity and high mortality [12] and signs often occur in the winter season. Most often, lesions are found in the laryngeal mucosa covering the arytenoid cartilage [12]. Microbiologically, a variable participation of *F. necrophorum, Bacteroides *spp., *Trueperella pyogenes, Streptococcus* spp*., Pasteurella* spp*.* and *Escherichia coli* has been reported [5, 7, 8, 10–12]. Often a mixed population of Gram-positive and Gram-negative bacteria is present [12].

The pathogenesis of the condition remains unknown. *Fusobacterium necrophorum* alone is not able to pass through intact mucosa; therefore a primary traumatization of affected tissue is being considered as an initiating event [4, 8, 11]. Irritating or toxic compounds, coughing as well as aspirated grains may cause primary mucosal damage leading to disruption of the mucosal barrier with secondary bacterial colonization [4, 5, 8, 12]. Also, the influence of male sexual hormones leading to an edematous swelling of the mucous membranes could be a factor in the development of the disease in males [13]. Additionally, a breed predisposition is discussed in Texel and Southdown sheep [7, 8, 12]. This might result from variations in laryngeal shape [7, 13, 14]. In a study comparing 43 larynges from Texel and Bluefaced Leicester rams, differences in the anatomy of the larynges between these breeds were shown. The short-headed Texel rams had for example a significant shorter and narrower larynx than the Bluefaced Leicester rams as well as a disproportional large epiglottis and arytenoid cartilage. Furthermore, the trachea of the Texel rams had a very narrow lumen described as funnel-shaped [14]. The pulmonary lesions are probably secondary changes either due to airborne colonization of pulmonary parenchyma by bacteria from the larynx or caused by dysfunction of the laryngeal closure with consecutive aspiration pneumonia. However, secondary pneumonia is rarely reported in ovine laryngitis [12].

Laryngeal lesions, comparable to those described in sheep, have been reported in calves [15], camelids [16], a white-tailed deer (*Odocoileus virginianus*) [17], thoroughbreds [18] and humans [19]. In calves, similar lesions are part of oral necrobacillosis, which is caused by a monoinfection with *F. necrophorum* [15]*.* In racing horses these lesions are detected in arytenoid chondropathy [4]. In both diseases, the pathogenesis remains unclear although mucosal traumatization due to forced respiration and drenching is considered to be a factor predisposing to bacterial colonization [4]. In humans an auto-immune pathogenesis as well as *Mycobacterium tuberculosis* or *Corynebacterium diphtheriae* induced lesions are discussed [19, 20].

Ovine laryngeal chondritis is associated with a poor prognosis. Therapy with antibiotics and corticosteroids can cure the disease in very early stages, but often fails [5, 7, 8]. In case of life-threatening conditions, a tracheostomy may help to avoid the death of the animal [13].

Differential diagnoses include swellings of lymph nodes (for example due to pseudotuberculosis), foreign bodies, ovine pulmonary adenocarcinoma or Maedi [13]. To prevent laryngeal chondritis, a sheep once suffering from the disease should not be used for breeding if it survives as a hereditary component cannot be excluded. Additionally, oral applications should be carried out carefully to avoid traumatization.

In conclusion, albeit being a rare disease, laryngeal chondritis should be considered as a differential diagnosis in sheep with upper airway disease, also in mainland Europe.

## Data Availability

Data sharing is not applicable to this article as no datasets were generated or analysed during the current study.
